# Comparative Effects of Intra-Articular versus Intravenous Mesenchymal Stromal Cells Therapy in a Rat Model of Osteoarthritis by Destabilization of Medial Meniscus

**DOI:** 10.3390/ijms242115543

**Published:** 2023-10-24

**Authors:** Felipe Bruno Dias de Oliveira, Eliane Antonioli, Olívia Furiama Metropolo Dias, Jean Gabriel de Souza, Sudha Agarwal, Ana Marisa Chudzinski-Tavassi, Mario Ferretti

**Affiliations:** 1 Hospital Israelita Albert Einstein, São Paulo 05652-900, Brazil; fbdoliveira@outlook.com (F.B.D.d.O.);; 2 Department of Biomedical Engineering, Wayne State University, Detroit, MI 48202, USA; jean.souza@wayne.edu; 3 CENTD Centre of Excellence in New Target Discovery, Butantan Institute, São Paulo 05503-900, Brazil; 4 Division of Rheumatology and Immunology, The Ohio State University College of Medicine, Columbus, OH 43210, USA; 5 Division of Biosciences, The Ohio State University College of Dentistry, Columbus, OH 43210, USA; 6 Laboratório de Desenvolvimento e Inovação, Butantan Institute, São Paulo 05503-900, Brazil; 7 Departamento de Ortopedia e Traumatologia, Escola Paulista de Medicina, Universidade Federal de São Paulo, São Paulo 04039-032, Brazil

**Keywords:** osteoarthritis, mesenchymal stromal cell, mesenchymal stem cell, intra-articular, intravenous, inflammation

## Abstract

Transplanted mesenchymal stromal cells (MSCs) exhibit a robust anti-inflammatory and homing capacity in response to high inflammatory signals, as observed in studies focused on rheumatic diseases that target articular cartilage (AC) health. However, AC degradation in osteoarthritis (OA) does not necessarily coincide with a highly inflammatory joint profile. Often, by the time patients seek medical attention, they already have damaged AC. In this study, we examined the therapeutic potential of a single bone marrow MSC transplant (2 × 10^6^ cells/kg_bw_) through two different routes: intra-articular (MSCs-IAt) and intravenous (MSCs-IVt) in a preclinical model of low-grade inflammatory OA with an established AC degeneration. OA was induced through the destabilization of the medial meniscus (DMM) in female Wistar Kyoto rats. The animals received MSCs 9 weeks after surgery and were euthanized 4 and 12 weeks post-transplant. In vivo and ex vivo tracking of MSCs were analyzed via bioluminescence and imaging flow cytometry, respectively. Cytokine/chemokine modulation in serum and synovial fluid was measured using a multiplex panel. AC degeneration was quantified through histology, and hindlimb muscle balance was assessed with precision weighing. To our knowledge, we are the first group to show the in vivo (8 h) and ex vivo (12 h) homing of cells to the DMM–OA joint following MSCs-IVt. In the case of MSCs-IAt, the detection of cellular bioluminescence at the knee joint persisted for up to 1 week. Intriguingly, intra-articular saline injection (placebo-IAt) resulted in a worse prognosis of OA when compared to a non-invasive control (placebo-IVt) without joint injection. The systemic cytokines/chemokines profile exhibited a time-dependent variation between transplant routes, displaying a transient anti-inflammatory systemic response for both MSCs-IVt and MSCs-IAt. A single injection of MSCs, whether administered via the intra-articular or intravenous route, performed 9 weeks after DMM surgery, did not effectively inhibit AC degeneration when compared to a non-invasive control.

## 1. Introduction

According to the World Health Organization, OA is the second most prevalent musculoskeletal disease worldwide [1]. OA is characterized as a disorder of the whole joint, featuring synovial inflammation, AC degeneration, subchondral bone erosion, the narrowing of joint space, osteophyte formation, joint pain, and stiffness, ultimately leading to functional impairment [2]. Currently, there is no non-surgical treatment that can promote AC repair in OA. Consequently, disease progression often culminates in total joint arthroplasty [3].

Proinflammatory signals play an important role in OA coordinating the influx of immune cells to sites of injury, initiating tissue damage [4]. In this context, the therapeutic potential of MSCs has been investigated [5,6]. MSCs are multipotent cells with immunomodulatory capacity. Through cell–cell or cell–tissue interactions, they can downregulate proinflammatory signals induced by cytokines, suppress inflammation, and initiate tissue repair [7,8]. This capacity is linked to MSCs’ communication with macrophages, as they attract pro-inflammatory M1 macrophages and subsequently induce their polarization to a pro-resolutive, immunosuppressive M2 state. By doing so, MSCs initially aid in the removal of debris and dead cells, and later create a favorable environment for tissue repair [8,9].

MSC-based treatment via intravenous and intra-articular routes demonstrated to be effective in attenuating inflammation in mouse models of rheumatoid arthritis (RA). In these models, MSCs elicited systemic immune tolerance and homed to the injured joint [10]. However, OA typically exhibits a lower-grade inflammatory profile compared to RA [11].

Studies reporting positive effects of MSCs-IAt treatment on AC typically rely on inflammatory-driven OA animal models induced by joint enzymatic injections, severe joint destabilization surgery, or MSC transplantation during the initial inflammatory response caused by the model induction [5]. However, fewer studies have explored MSCs-IVt for OA treatment [12,13,14,15,16,17,18], and the ones reporting beneficial effects on AC have also used similar approaches as MSCs-IAt studies [13,14,16,17].

The delay in MSCs injection following OA model induction appears to reduce the transplant efficiency for AC treatment [19,20,21]. However, OA is a chronic, low-grade inflammatory disease [11], characterized by limited diagnostic approaches. In general, when patients seek medical care, they already have joint impairment and advanced AC degeneration [22]. Therefore, selecting an animal model that closely resembles the disease’s pathogenesis and progression, along with a strategically timed treatment, has translational implications for OA therapy [23,24].

For the first time, we compared the therapeutic potential of IAt and IVt routes of bone marrow MSCs in low-grade inflammatory OA. More importantly, we utilized a well-established model (DMM) to provoke a natural-like progression of OA [25] over a period of 9 weeks before treatment, and an individualized number of MSCs per transplant.

## 2. Results

### 2.1. Localization of MSCs in DMM–OA-Afflicted Knees When Delivered by IAt or IVt

**Figure 1:**
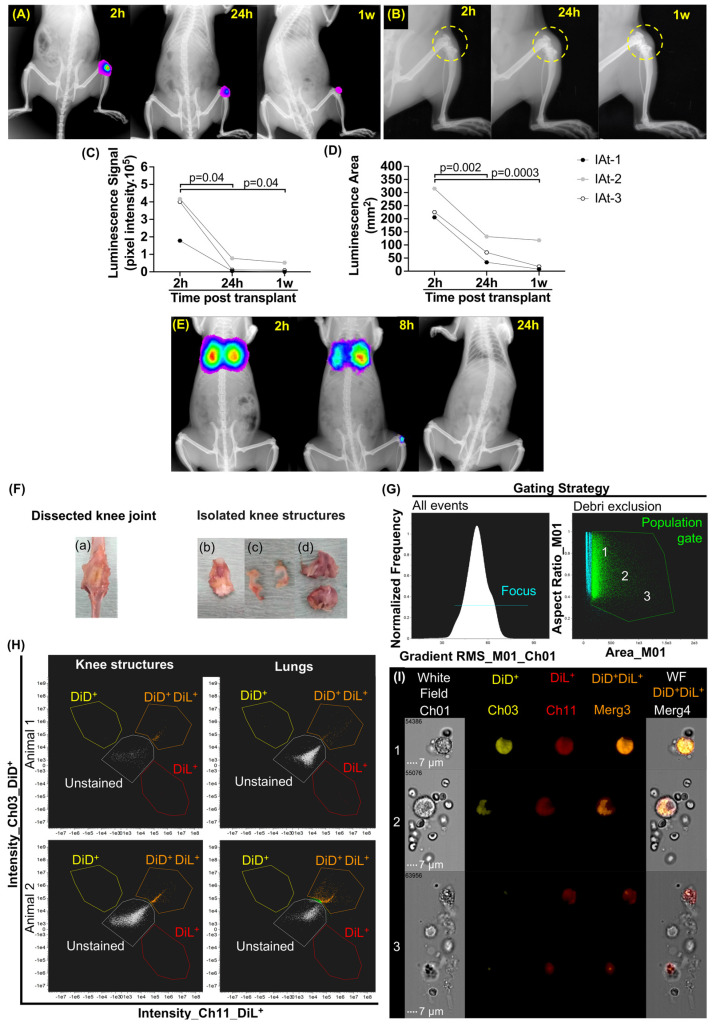
In vivo bioluminescence tracking of mesenchymal stromal cells (MSCs) at two (2 h), eight (8 h), and 24 hours (24 h), up to one week (1 w) after transplantation, along with ex vivo identification of double positive (DiL^+^ DiD^+^) MSC events through imaging flow cytometry (IFC), conducted at 12 h after intravenous transplantation. (**A**) Intra-articular transplant (IAt) of MSCs, and cellular bioluminescent signal represented in rainbow scale. (**B**) Knee joint (yellow dashed circle) and swelling after IAt. (**C**,**D**) Quantification of pixel intensity and luminescence area for IAt. (**E**) Intravenous transplant (IVt) in vivo of MSCs, and cellular bioluminescent signal represented in rainbow scale [26]. (**F**) Dissected knee joint: (**a**) anterior view; (**b**) knee structures containing synovial membrane, Hoffa fat pad, patella, and patellar tendon; (**c**) menisci; (**d**) femur and tibial cartilage with attached subchondral bone. (**G**) Gating strategy: population gate (green line) and image sampling zones 1, 2 and 3. (**H**) Dot plot of double positive events (orange dots) in lungs and DMM–OA-pooled knee structures. (**I**) Images of labeled cell morphology inside the gate according to zones 1, 2 and 3 (60× objective lens). White dotted line: 7 μm scale bar. IFC channels of labeled MSCs: DiD^+^ (yellow); DiL^+^ (red); DiL^+^ and DiD^+^ merge (orange). Brackets indicate comparisons and the respective *p*-values.

We employed syngeneic cells derived from bone marrow, demonstrating multipotent differentiation ability and the expression of MSCs’ specific cell surface markers (Figure S1A–D). These cells were transfected to express luciferase transgene (Figure S2A,B). Initial pilot studies were conducted to assess in vitro bioluminescence efficiency and D-luciferin/luciferase kinetics in vivo to select analysis time points following IAt and IVt (Figure S2C,D). After these studies, we were able to demonstrate that following IAt (2 × 10^6^ cells/kg_bw_), were viable bioluminescent MSCs remained in the DMM–OA lesion for up to one week (N = 3). However, the luminescent area (mm^2^) decreased by 69.91 ± 12.77% within 24 h and 83.71 ± 18.32% after one week when compared to a 2 h post-transplantation time point (Figure 1A,C,D). In parallel, joint swelling observed at 24 h (2 h: 3.93 ± 0.18 mm vs. 24 h: 5.23 ± 0.60 mm; *p* = 0.034) also subsided after one week (24 h: 5.23 ± 0.60 mm vs. 1 w: 4.46 ± 0.72 mm; *p* = 0.016) (Figure 1B and Figure S3). Interestingly, following IVt (4 × 10^6^ cells/kg_bw_), the bioluminescent MSCs localized at the DMM–OA-afflicted knee joints, as well as lungs, within 8 h in one animal (N = 3), and were undetectable at both sites by the 24 h mark (Figure 1E). Since the non-detection of cells could be attributed to the lower bioluminescence intensity, we confirmed IVt studies via the membrane labeling of MSCs (4 × 10^6^ cells/kg_bw_) with DiL and DiD, and detecting them using IFC (Figure 1F–I and Figure S4A,B). We attempted to localize double positive MSCs at the DMM–OA-afflicted knees by dissecting knee structures. These structures were pooled and digested to isolate cells for further analyses. Due to the greater sensitivity of the IFC scanning and gating strategy (Figure 1G and Figure S4A,B), we were able to detect double positive MSCs (DiL^+^ DiD^+^) at the DMM–OA-afflicted knees and in lungs up to 12 h post-IVt (Figure 1H). Images of double positive events inside the gate ranged from whole cells to cellular debris (Figure 1I).

### 2.2. Induction of Systemic Cytokines/Chemokines in DMM–OA-Afflicted Rats

**Figure 2:**
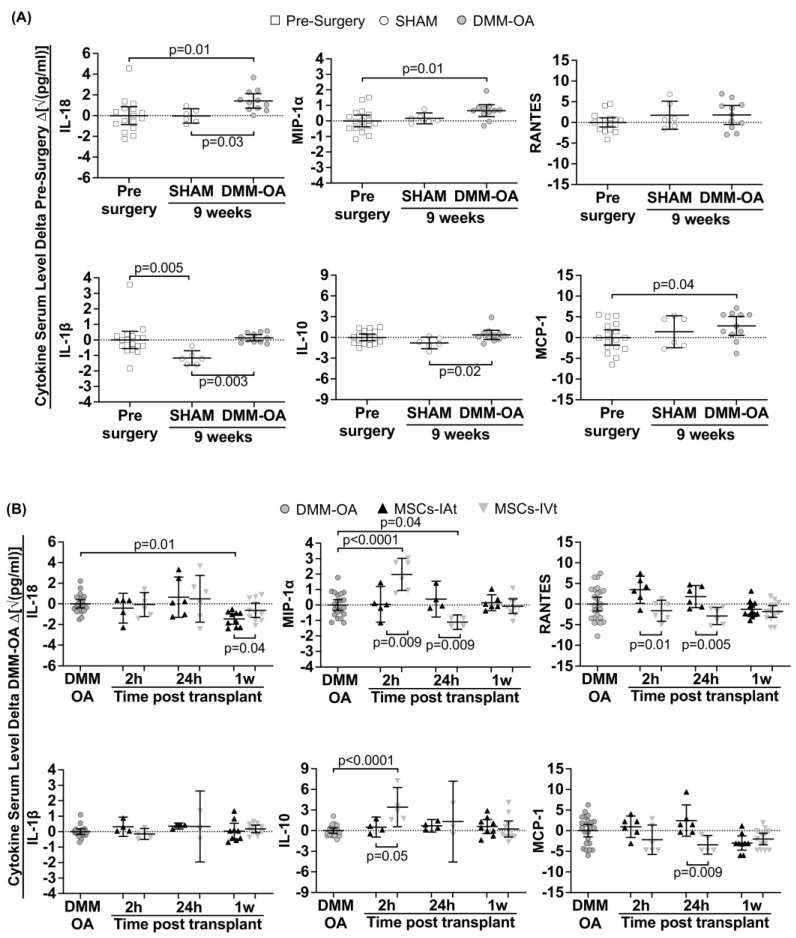
Animal model immunomodulation at pre-surgery and 9 weeks post-surgery, and acute immunomodulation at two (2 h) and 24 h (24 h), up to one week (1 w) after mesenchymal stromal cells (MSCs) intra-articular (IAt) or intravenous (IVt) transplantation. The serum levels of cytokines/chemokines were measured using a multiplex bead assay. The horizontal dotted line represents the average serum levels in the control group for two specific time points: (**A**) pre-surgery; (**B**) 9 weeks post DMM–OA surgery. The data are expressed as the delta between the control and intervention groups. Brackets indicate comparisons and the respective *p*-values.

Initially, we examined the presence of ten major immunomodulatory cytokine/chemokines in serum samples at various time points: pre-surgery, nine weeks after SHAM surgery, and nine weeks after DMM–OA induction (Figure 2A). Among these cytokines, TNF-α, IL-6, IL-13, and IL-4 were undetectable. However, when compared to pre-surgery levels, serum levels of IL-18, MCP-1, and MIP-1α were significantly upregulated in DMM–OA, while IL-1β, IL-10, and RANTES remained unaffected. Importantly, in the SHAM group, the levels of these cytokines/chemokines were similar to those observed in the pre-surgery samples, with the exception of IL-1β and IL-10, which were lower than in the DMM–OA.

### 2.3. Short-Term Regulation of Systemic Cytokines/Chemokines by Transplanted MSCs

MSCs, whether delivered through IAt or IVt routes, displayed a distinct regulation of systemic cytokines/chemokines, depending on the type of cytokine and the post-transplant time point. In this section, we compared serum cytokines levels in two contexts: (1) between DMM–OA rats treated with MSCs-IAt or MSCs-IVt to evaluate the ability of MSCs to modulate cytokines, and (2) to assess the relative effectiveness of IAt and IVt treatment modalities (Figure 2B). Systemic levels of IL-18 and IL-1β remained largely unchanged at 2 or 24 h, regardless of the treatment route. However, at the 1 week mark, suppression of IL-18 was significantly more pronounced in response to MSCs-IAt compared to MSCs-IVt treatment. MIP-1α and IL-10 levels showed no significant variation in serum samples from rats treated with MSCs-IAt at 2, 24 h, or 1 week post application, in comparison to the DMM–OA group. In contrast, following MSCs-IVt, both MIP-1α and IL-10 experienced rapid and significant upregulation compared to DMM–OA, as well as to MSCs-IAt treatment. However, these levels returned to baseline within one week following MSCS-IAt and MSCs-IVt treatment, in comparison to the DMM–OA group. Moreover, MIP-1α was significantly downregulated after 24 h of MSCs-IVt treatment. RANTES and MCP-1 levels remained largely unaltered by any of the treatments when compared to the DMM–OA group. Notably, we observed lower levels with MSCs-IAt treatment compared to MSCs-IVt at 2 and 24 h for RANTES and 24 h for MCP-1.

### 2.4. Long-Term Systemic and Local Regulation of Cytokines by Transplanted MSCs

**Figure 3:**
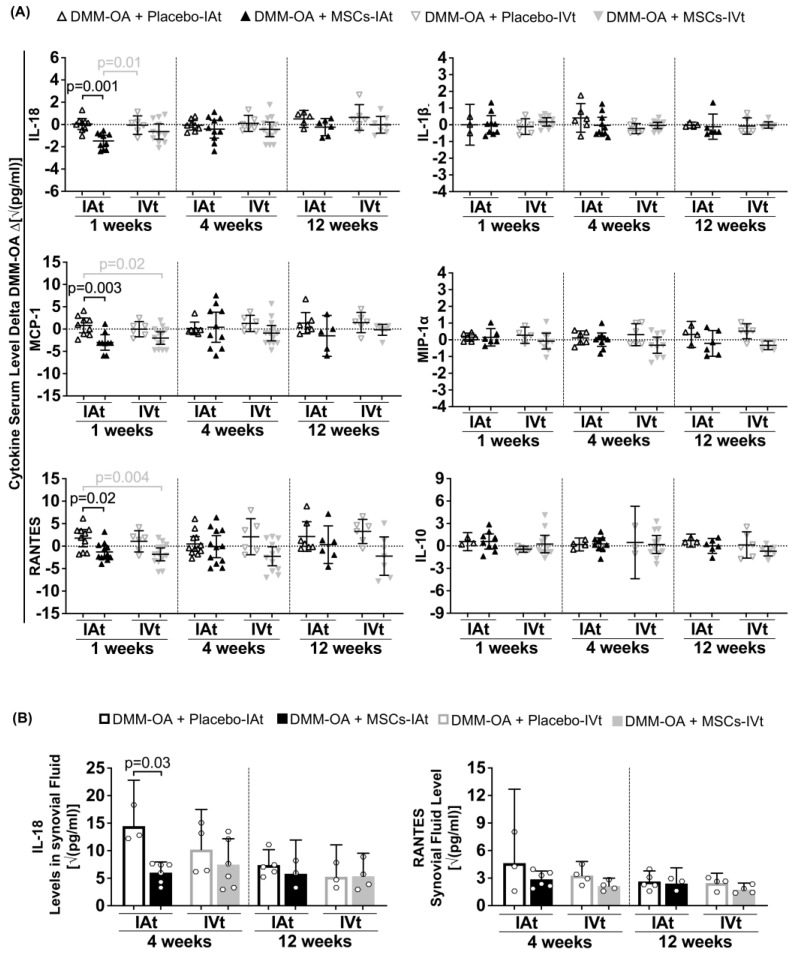
Long-term immunomodulation at one (1 w), four (4 w), and 12 weeks (12 w) after mesenchymal stromal cells (MSCs) intra-articular (IAt) or intravenous (IVt) transplantation. (**A**) Serum and (**B**) synovial fluid levels of cytokines/chemokines measured using a multiplex bead assay. For serum analysis, the horizontal dotted line represents the average serum level of each group average at 9 weeks after DMM–OA surgery (positive control). The data are expressed as the delta between the positive control and intervention groups. Vertical dashed lines separate comparisons at different time points. Black brackets indicate comparisons between IAt vs. IAt or IVt vs. IVt, while grey brackets represent comparisons between IAt vs. IVt. Statistical *p*-value is shown above the respective bracket.

The modulation of serum cytokines/chemokines was primarily observed at the 1-week time point. In response to MSCs-IAt treatment, the serum levels of IL-18, MCP-1, and RANTES were lower when compared to placebo-IA. Additionally, when compared to placebo-IVt, the IL-18 level was lower in response MSCs-IAt. There were no differences between MSCs-IVt and placebo-IVt treatments at any time point. However, when compared to placebo-IAt, MCP-1 and RANTES were significantly lower in response to MSCs-IVt. No differences were observed between treatment groups for IL-1β, MIP-1α, and IL-10 serum levels at any time point (Figure 3A). Subsequently, an analysis of synovial fluids revealed that, following MSCs-IAt treatment, IL-18 was significantly lower at 4 weeks when compared to placebo-IAt. However, no differences in IL-18 at 12 weeks and RANTES at 4 and 12 weeks were observed in response to MSCs-IAt or IVt (Figure 3B). To investigate the effect of MSC treatment in the absence of cartilage degradation, we also analyzed rats that received SHAM surgery and MSCs. As expected, no significant differences in cytokine/chemokine levels were observed between MSCs-IAt and MSCs-IVt in serum specimens or synovial fluids in SHAM groups (Figure S5A,B).

### 2.5. Changes in Joint Cartilage in Response to MSC Treatment

**Figure 4:**
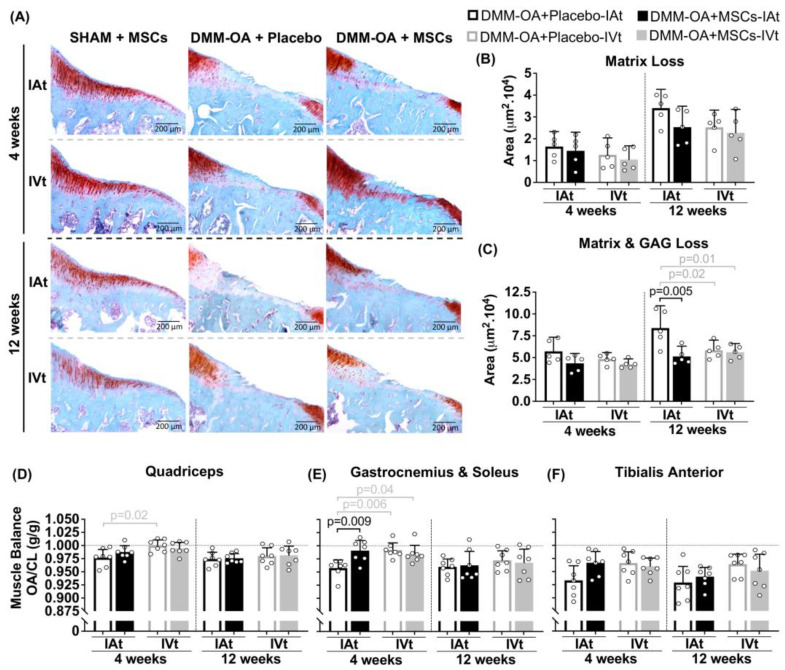
Cartilage and muscle balance analysis after mesenchymal stromal cells (MSCs) intra-articular (IAt) or intravenous (IVt) treatment. (**A**) Safranin-O cartilage glycosaminoglycan staining of the medial tibial plateau for histological representation (4× objective lens). (**B**,**C**) Scoring of cartilage injury (SHAM groups as negative controls not included in the analysis). (**D**–**F**) Muscle balance ratio for quadriceps, gastrocnemius, and soleus and tibialis anterior between the DMM–OA limb joint and the contralateral limb (CL). Horizontal dotted line represents perfect muscle balance ratio. Vertical dashed lines isolate timepoint comparisons. Black brackets indicate IAt vs. IAt or IVt vs. IVt comparisons, while grey brackets represent IAt vs. IVt comparisons. Statistical *p*-values are shown above the respective bracket.

To assess the impact of the surgical procedure alone, SHAM groups were included to evaluate joint damage caused by the surgery itself. As expected, a histological analysis of knee cartilage from rats that underwent SHAM surgeries, followed by either IAt or IVt MSC treatment, revealed no focal matrix or GAG loss (Figure S6A,B) at 4 and 12 weeks. In contrast, knees afflicted by DMM–OA demonstrated matrix and GAG loss at 4 and 12 weeks. While there were no detectable differences between treatments in terms of matrix loss, when adjacent proteoglycan loss was considered, MSCs-IAt showed less cartilage degeneration at 12 weeks compared to placebo-IAt. There were no differences between MSC treatments or between MSCs-IVt and placebo-IVt. However, both MSCs-IVt and placebo-IVt exhibited less cartilage degeneration at 12 weeks compared to placebo-IAt (Figure 4A–C). No visible signs of synovial lining hyperplasia were observed in any surgery or treatment group (Figure S7).

### 2.6. Changes in Hindlimbs Muscle Balance Ratio

The ratio between DMM–OA and the contralateral leg’s muscle weight was analyzed as an indirect measure of limb functionality. At both 4 and 12 weeks, there were no significant differences between MSCs-treated groups and the placebo groups in terms of quadriceps muscle balance. However, it is worth noting that placebo-IAt showed a lower balance than placebo-IVt up to the 4 week mark. In the case of gastrocnemius and soleus muscles, MSC-IAt group exhibited a greater balance compared to placebo-IAt. Additionally, placebo-IVt and MSCs-IVt also demonstrated greater balance than placebo-IAt at 4 weeks but not 12 weeks (Figure 4D,E). No significant differences were observed in the tibialis anterior muscle (Figure 4F) or SHAM groups at 4 or 12 weeks (Figure S6D–F).

## 3. Discussion

In this study, we allowed rats to develop OA for 9 weeks after the DMM surgery before receiving MSC transplants. It is known that in the DMM–OA model, significant gene modulation occurs during the first 4 weeks post-surgery. However, around the 8-week mark, genes related to cell proliferation and extracellular matrix remodeling stabilize [27]. Major changes in articular cartilage and subchondral bone plate also occur at similar time points [28]. By allowing the model to develop, we aimed to avoid the initial high inflammatory response related to the surgery itself. This approach enabled the MSC treatment to focus on the pathophysiological aspects of DMM–OA, which closely resemble the slower, more chronic development of OA observed in clinical scenarios.

The number of cells used in MSC treatment for OA varies widely between studies, particularly for IAt interventions. In clinical trials, the cell numbers typically range from approximately 0.1–2.1 × 10^6^ cells/kg_bw_ for a patient weighing 70 kg [6]. In rat models of OA, the cell numbers used range from about 0.5–25 × 10^6^ cells/kg_bw_ for a 200 g animal [5]. Using an extremely high number of cells is impractical from a clinical perspective. To ensure transability and safety, we decided to use 2 × 10^6^ cells/kg_bw_., a cell number used in clinical settings for MSCs-IVt [29], and within the range used for MSC-IAt in OA patients. This allowed for meaningful comparisons between transplant routes.

Differences in bloodstream pharmacokinetics between MSCs-IAt and MSCs-IVt have been described previously in healthy animals [30]. Similar to earlier studies, we observed a significant loss of MSC bioluminescence signal 24 h after IAt, which progressively diminished within 1 week [30,31,32]. It has been observed that one day after MSCs-IAt, many cells are found in clusters in the synovial fluid [32]. However, whether anoikis contributes to the loss of signal as a cell death mechanism for cells that did not engraft inside the joint remains unclear and requires future investigation.

In order to enhance the detection of cells for the evaluating homing after IVt, we increased the number of cells for this analysis (4 × 10^6^ cells/kg_bw_). We found detectable MSC bioluminescence at the DMM–OA knee joint 8 h after IVt, but no signal was detected after 24 h. To the best of our knowledge, this is the first study to show in vivo systemic homing of MSCs to the injured knee in a low-grade inflammatory OA model caused by DMM surgery. We further confirmed these data using IFC and showed that MSC events detected in the DMM–OA knee structures following IVt ranged from whole cells to cell debris or phagocytosed-like cells. Whether these cells migrate inside the joint or engraft in surrounding tissues is a subject for future studies.

Consistent with our finding, a pilot study conducted on dogs with naturally occurring elbow OA found labeled MSCs in synovial fluid aspiration 24 h after IVt [12]. In vivo homing of MSCs was also detected 24 h following non-surgical post-traumatic induction of knee OA by mechanical ACL rupture [33]. However, in that study, MSCs-IVt was performed immediately after the procedure, which is known to elicit inflammatory response within the first day [34]. Similarly, in a case study involving a dog with ACL rupture and synovitis, in vivo homing was observed 6 h after MSC-IVt [35]. Histological evidence also supports labeled MSCs within knee joint structures following IVt [13,16]. In these studies, cells were transplanted right after a focal chondral defect surgical procedure [13] or seven days post intra-articular mono-iodoacetate injection [16], a chondrotoxic compound that causes a high inflammatory response up to one week after injection [23].

Serum levels of IL-18, MCP-1 (CCL2) and MIP-1α (CCL3) were slightly higher in DMM–OA compared to pre-surgery. These cytokines are found in patients’ sera and are potential biomarkers of OA [36,37,38]. MCP-1 and MIP-1α also have the capacity to chemoattract MSCs [39]. These chemokines are expressed by OA chondrocytes and synovial cells, and both have been implicated in the progression of OA induced by DMM surgery [40,41,42]. Given that DMM–OA is a focal injury model, one reason for the changes in serum levels of these chemokines may be their increased expression by the cells within the articular joint, which could play a role in stimulating the homing of MSCs to the DMM–OA knee following IVt.

The significant increase in MIP-1α in the serum 2 h after MSCs-IVt is characteristic of macrophage activity driven by the accumulation of transplanted cells in lung alveolar capillaries [43,44]. IL-10, an anti-inflammatory cytokine, is also produced by macrophages, TH1, TH2 cells, and other immune system components [45]. This abrupt regulation in both molecules indicates a systemic positive feedback loop mechanism aimed at regulating macrophages and maintaining tissue homeostasis [43,44,45]. In OA patients, lower levels of serum IL-10 has been associated with a higher joint radiographic severity [46]. An increase in systemic IL-10 levels could be beneficial for OA. However, the extent of change in this cytokine required to have a positive impact on treatment remains uncertain, as no differences were observed in muscle tissue or articular cartilage between the MSCs-IVt and placebo-IVt group.

RANTES (CCL5) is a chemokine responsible for attracting leukocytes to the injury site [47], while macrophages or monocytes [48] are primarily attracted by MCP-1. On the other hand, IL-18 belongs to the pro-inflammatory cytokine IL-1 family and is mainly produced by macrophages, although it can also be produced by other immune and non-immune cells [49]. The differences in short-term regulation of these molecules between MSCs-IAt and MSCs-IVt, occurring simultaneously with knee joint swelling, could be related to local immunomodulatory effects of MSCs-IAt during the induction and resolution of inflammation in the tissue repair process [8].

Furthermore, MSCs-IAt also demonstrated the ability to systemically (1 week) and locally (4 weeks) suppress IL-18 in comparison to placebo-IAt. IL-18 is present in the sera and synovial fluid from knee OA patients and is expressed by OA chondrocytes and synoviocytes. It is known to inhibit proteoglycan synthesis, thus contributing to cartilage degradation [36,49,50].

Moreover, while a single injection of MSCs did not inhibit matrix loss in any group, a positive effect of MSCs-IAt was observed in total cartilage degeneration and gastrocnemius and soleus muscle balance compared to placebo-IAt for up to 4 weeks. It is known that locally transplanted MSCs can engraft at the joint-surrounding tissues (e.g., muscle) [30,51] and have a positive effect in reducing muscular atrophy [52], which is a risk factor for OA development [53]. Therefore, it is plausible that the transient downregulation of IL-18, along with an improved muscle balance ratio during the initial weeks post-transplant contributes to the reduction in AC degeneration in the subsequent weeks.

A previous study showed that the effect of MSCs-IAt on OA is transient, and periodic injections were more effective than a single injection in reducing disease progression at 8 and 12 weeks, as compared to placebo-IAt, in a rat model of anterior cruciate ligament transection (ACLT) [31]. The difference in the effects of a single MSCs-IAt injection, as observed in our study, might be attributed to variations in study design, particularly in terms of OA model progression and severity characteristics. The slower disease development in the DMM–OA model may have allowed the detection of more subtle AC changes. Additionally, a recent short-term study of MSCs-IVt period injections (4 weeks) did not show changes in AC. However, gait performance improved, potentially due to a systemic immunomodulatory effect, as indicated by the suppression of serum inflammatory markers (MCP-1, TNF, and PG(E)_2_), according to the authors [18]. Collectively, these findings suggest that due to the transient nature of MSCs’ effects, therapies aiming to improve AC health in OA may be more effective with long-term protocols and periodic injections, regardless of transplant route.

In our study, we employed the placebo-IVt as a non-invasive transplant control, meaning it involved no joint injection, to compare with MSC treatment routes and placebo-IAt. It appeared that knee placebo-IAt exacerbated short-term inflammatory signals and led to long-term AC degeneration and unfavorable muscle balance in relation to the DMM–OA model. In contrast, some clinical trials have reported positive effects of intra-articular saline injections for OA [54]. However, it is important to note that these clinical studies rely on patient-reported outcomes, primarily the assessment of self-perceived pain and function. The placebo effect in OA can stem from various non-biological factors, including patient education about OA, an increased awareness of the disease, and patient perception and expectations regarding the novel treatment. Also, in some studies, a synovial fluid aspiration is conducted before the intra-articular intervention, which temporarily reduces inflammatory molecules within the joint.

Intra-articular injection per se is among the most invasive non-surgical intervention for OA and may, to some extent, inflict damage on the joint. In animal models, a high injection volume (100 μL) can directly compromise joint integrity [55]; however, even a relatively small injection volume, such as 20 μL, which is considered a “safe” volume, can trigger a significant local inflammatory response and muscle weakness in healthy rats after intra-articular intervention [56]. It is plausible that the degree of invasiveness of intra-articular injections differs between humans and small animal models. Therefore, when making translational comparisons, it is crucial to exercise caution. Nevertheless, our study underscores the importance of including a non-invasive control in experimental designs where IAt is chosen as the treatment delivery route. This consideration is important because the placebo joint injection may be amplifying differences in outcome measures between the treatment and control groups.

For SHAM + MSCs groups, both IAt and IVt were equally important to analyze possible negative outcomes related to cellular transplant per se in different delivery routes. While we did not observe any noticeable differences, the absence of SHAM + placebo groups prevented a more comprehensive understanding of these outcomes. Finally, we would like to clarify that despite the DMM low-grade inflammatory profile of this model, it is possible that sample collection or manipulation of serum and synovial fluid could have influenced the non-detection of some analytes from the multiplex panel, which impaired their group comparisons. Therefore, the low levels observed of these cytokines should not be interpreted as the lack of expression in similar experimental designs.

## 4. Materials and Methods

### 4.1. Animals

Wistar Kyoto rats (females; age: 6.65 ± 1.02 months; weight: 241.45 ± 11.52 g) were utilized for this study (treatments: N = 7/group; bioluminescence: N = 4/transplant route; imaging flow cytometry: N = 2; MSCs isolation: N = 6; total: N = 100). Rats were housed in a controlled environment, with temperature between 24 and 26 °C, 40–50% humidity, 12/12 h light/dark cycles, and ad libitum food/water. The animal facility “Centro de Experimentação e Treinamento em Cirurgia” at “Instituto Israelita de Ensino e Pesquisa Albert Einstein” holds accreditation from the “Association for Assessment and Accreditation of Laboratory Animal Care (AAALAC)”. All procedures (study design, surgical procedure, administration of cells and imaging, number of animals) were conducted in compliance with the approved protocols of the Hospital Israelita Albert Einstein’s Ethics Committee for experimentation and animal use (number: 2275-15). At the conclusion of the experiments, the animals were euthanized under 5% isoflurane followed by CO_2_ asphyxia or ketamine/xylazine overdose.

### 4.2. Animal Model of OA

OA was induced in the right knee using the DMM model, following previously described methods [25]. Briefly, a skin incision was made medially to the patellofemoral ligament, the patella was then laterally dislocated, and the Hoffa adipose tissue was bluntly dissected to expose the medial meniscotibial ligament. Subsequently, the ligament was transected, and the knee was sutured. For SHAM surgery, the exact same procedure was carried out, with the exception that, after visualization of the medial meniscotibial ligament, the joint was closed without transecting the ligament. A nine-week post-surgery interval was considered as sufficient for the establishment of OA. Anesthesia and pain monitoring are detailed in Methods S1.

### 4.3. MSC Isolation, Characterization and Differentiation

MSCs were isolated from bone marrow of femur and tibia of female Wistar Kyoto rats (Figure S1A). Bone marrow was harvested by centrifuging bones via cut epiphyses at 500 × rcf for 3 min. The cells were treated with a lysis buffer (155 mM NH_4_Cl, 12 mM NaHCO_3_, and 0.1 mM EDTA) and were then washed twice with cold phosphate-buffered saline (PBS) to eliminate erythrocytes. Subsequently, cells were suspended in α-modified minimum essential medium (α-MEM), supplemented with 20% fetal bovine serum (FBS), 1% penicillin–streptavidin, and 1% L-glutamine (Gibco^®^, Thermo Fisher Scientific, Waltham, MA, USA). They were cultured in an incubator at 37 °C and 5% CO_2_. After 24 h of plating, adherent cells were washed 3 times with cold PBS, trypsinized (0.025% trypsin/0.1% EDTA/sodium pyruvate), plated at 5000 cells/cm^2^, and used between P8 an P12. FBS was reduced to 10% after P5. MSCs at P8 were characterized using standard MSCs’ markers: CD90, CD105, CD29, CD44, CD34, CD45, and CD11b/c. The cellular populations were acquired via flow cytometry (BD LSRFortessa^TM^, Becton, Dickinson and Company, Franklin Lakes, NJ, USA) and data were analyzed using the FlowJo^TM^ v.10 (Becton, Dickinson and Company, Franklin Lakes, NJ, USA) software. The multipotency of MSCs in differentiating into osteocytes, adipocytes, and chondrocytes lineages was assessed between P8 and P12 using StemPro^®^ Differentiation kits: A10072-01, A10070-01, and A10071-01 (Gibco^®^, Thermo Fisher Scientific, Waltham, MA, USA), respectively. The differentiation was confirmed by staining adipocytes with Oil Red, chondrocytes with Alcian Blue, and osteocytes with Alizarin Red. Aliquots containing 2.5 × 10^5^ cells in 100 µL of PBS were incubated with single-conjugated antibody according to manufacturer’s instructions (abcam^®^, Cambridge, UK). The cytometer acquisition protocol was set to 10,000 events. One unstained aliquot was used for cell autofluorescence control (FITC, PE, and APC fluorochrome channels). It was also used to set the population gate based on cell size (side scatter—SCC) and granularity (forward scatter—FSC). Standard expression of positive and negative MSC markers was considered as ≥95% for CD90, CD105, CD29, CD44 and ≤5% for CD34, CD45, CD11b/c, respectively (Figure S1B,C).

### 4.4. Luciferase Transgene Expression

MSCs were transfected with a puromycin-resistant luciferase transgene lentiviral vector (RediFect Red-Fluc-Puromycin; CLS960002, PerkinElmer^®^, Waltham, MA, USA), using a multiplicity of infection (MOI) of 20. To isolate MSCs-expressing luciferase, 48 h after transfection, the cells were incubated with 2 μg/mL puromycin for 6 days. Subsequently, luciferase activity was quantified using Spectramax^®^ M5e (Molecular Devices, San Jose, CA, USA) and bioluminescence imaging with Carestream In-Vivo MSFX Pro (Molecular Bioimaging, Bend, OR, USA) and the software Molecular Imaging v.7.5 (Bruker, Billerica, MA, USA) (Figure S2A,B).

### 4.5. MSC Transplant

For transplantation to animals, MSCs were harvested, counted, suspended in PBS, and kept on ice. A dose of 2 × 10^6^ cells/kg per body weight (bw) in 50 µL of PBS was administered medially to the knee patellar ligament for IAt injections. For IVt, the same number of MSCs were applied via caudal vein in a 1 mL PBS suspension. This cell quantity falls within the translational range for IVt [29]. Placebo transplants followed the same procedures, but with PBS without MSCs.

### 4.6. In Vivo Imaging

**Figure 5:**
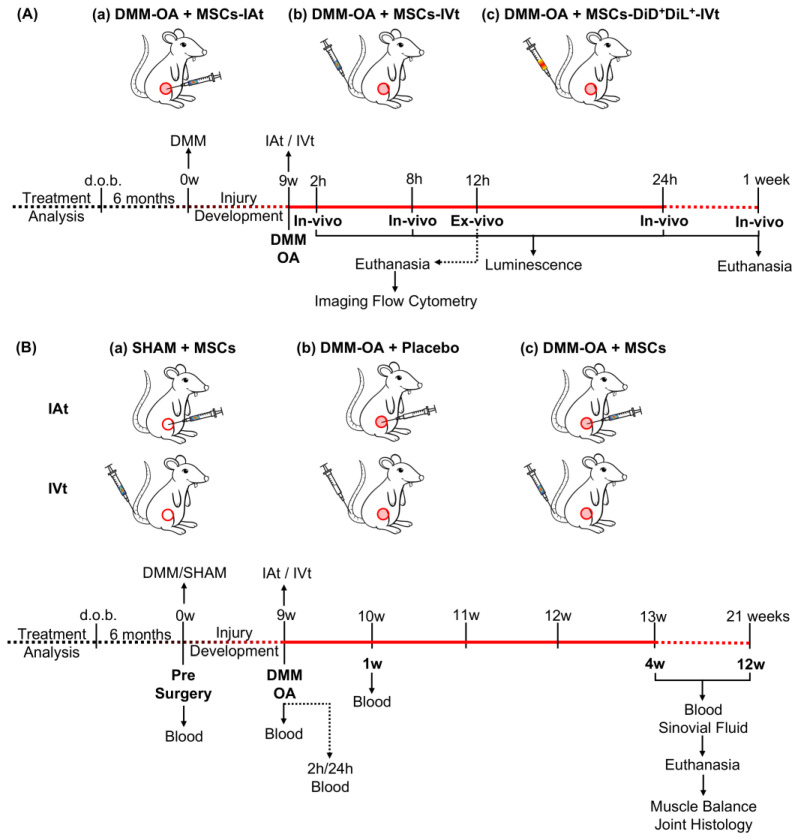
Experimental design. (**A**) To track the fate of transplanted MSCs in rats with DMM–OA-afflicted knees: (**a**) MSCs were intra-articularly transplanted (IAt) (2 × 10^6^ cells/kg per body weight (bw) in 50 µL PBS) administered medially to the knee patellar ligament; (**b**) MSCs (4 × 10^6^ cells/kg_bw_) were intravenously transplanted (IVt) via caudal vein and their homing to the knee with DMM–OA (the site of inflammation) was tracked via bioluminescence imaging at 2, 8, and 24 h and 1 wk. (**c**) In a separate experiment, MSCs (4 × 10^6^ cells/kg_bw_) labeled with DiL and DiD lipophilic tracers (Molecular Probes, Invitrogen^®^) were injected via IVt, and animals were euthanized 12 h post-IVt. Subsequently, joint and lung tissue were harvested to isolate MSCs, and their fluorescence was monitored via imaging flow cytometry. (**B**) Examination of the effect of MSCs administered via IAt or IVt on DMM–OA. (**a**) MSCs were administered via IAt or IVt in rats with SHAM surgery. (**b**) As placebo, PBS alone was administered via IAt or IVt in rats with DMM–OA. (**c**) MSCs were administered via IAt or IVt in rats with DMM–OA as a treatment. All rats were bled via gingival vein prior to DMM surgery, 9 weeks following the induction of DMM–OA, and 2 and 24 h and 1 week following IAt or IVt. Four and 12 weeks following IVt or IAt, all rats were bled, synovial fluid collected, they were euthanized, and muscle and joints harvested for analysis. Red filled circles: DMM surgery; red open circles: SHAM surgery.

Luciferase-transfected MSCs (2 × 10^6^ cells/kg_bw_) that were injected IAt into the knees affected by DMM–OA were tracked using bioluminescence at 2 and 24 h, and 1 week. Similarly, MSCs (4 × 10^6^ cells/kg_bw_)-administered IVt in DMM–OA rats were tracked at 2, 8, and 24 h and 1 week (Figure 5A(a,b)). The IVt cells’ number was increased to overcome assay detection limitations. In vivo bioluminescence and radiographic imaging were obtained with the Carestream In-Vivo MSFX Pro (Molecular Bioimaging, Bend, OR, USA), and the images were merged and analyzed using Molecular Imaging software v.7.5 (Bruker, Billerica, MA, USA). The bioluminescent signal capture was adjusted to 8x binning. Animals were intraperitoneally injected with D-luciferin (150 mg/kg_bw_ at 30 mg/mL), and images were captured 6 min later. The optimal time for bioluminescence was pre-determined through time-lapse imaging, with images taken every three minutes for 45 min (Figure S2C,D). Radiographic images were captured using the following parameters: focus 0.95; focus plane 10; X-ray exposition 2.9 s; lead filter of 0.8 mm photons/seconds/m^2^. Knee swelling following MSCs-IAt was measured by the diameter of the circumference delimited by the skin over the knee, distal femur, and proximal tibial epiphysis using radiographic images (Figure S3) and the ImageJ2 v.2.1.0/1.53c (https://imagej.net/) software.

### 4.7. Ex Vivo Imaging

As a complementary analysis to in vivo imaging, the same number of MSCs were labeled with DiL and DiD fluorescent Lipophilic Tracers (Molecular Probes^®^, Invitrogen^TM^, Thermo Fisher Scientific, Waltham, MA, USA) and injected via IVt into the DMM–OA animals (4 × 10^6^ cells/kg_bw_ in 1 mL PBS) (Figure 5A(c)). The animals were euthanized 12 h post-transplant, and tissue samples from the DMM–OA knee (the site of inflammation) were collected. At necropsy, the lungs, knee joint menisci, femur and tibial cartilage with attached subchondral bone, and anterior knee structures containing synovial membrane, Hoffa fat pad, patella, and patellar tendon were isolated. These tissues were then partially digested with 2 mg/mL Collagenase type IA (Sigma-Aldrich^®^, St. Louis, MO, USA) for one hour at 37 °C. The digested tissues were pooled, passed through a cell strainer (0.5 µm), washed with PBS, and their fluorescence was acquired using the imaging flow cytometer ImageStream^®X^ Mk II (Merck KGaA, Darmstadt, Germany). The data were analyzed via IDEAS^®^ 6.2 (Merck KGaA, Darmstadt, Germany) software (Gating strategy in Methods S2 and Figure S4A,B).

### 4.8. Treatment of Rat Knees Receiving DMM–OA or SHAM Surgery with MSCs or Placebo via IAt or IVt

The effect of IAt or IVt application of MSCs on knees afflicted with DMM–OA was evaluated at two different endpoints: 4 or 12 weeks following MSC transplant. For each time point, 6 groups were designed, and animals were assigned (not randomized/blinded): SHAM + MSCs-IAt, or SHAM + MSCs-IVt, (Figure 5B(a)); DMM–OA + placebo-IAt or DMM–OA + placebo-IVt (Figure 5B(b)); DMM–OA + MSCs-IAt or DMM–OA + MSCs-IVt (Figure 5B(c)).

### 4.9. Blood Specimen Collection

Blood samples were collected via gingival vein [57] at pre-surgery, 9 weeks DMM–OA, 1, 4, and 12 weeks after MSCs transplantation for all groups. Additional blood samples were collected at 2 and 24 h for DMM–OA + MSCs-IAt and IVt groups. After allowing the blood to clot for 25–30 min, it was centrifuged for 5 min at 10,000 rcf. The sera were collected and stored at −80 °C for up to one year. Before analysis, the samples were thawed at room temperature, thoroughly mixed by vortexing, and centrifuged for 5 min × 1000 rcf, to remove any particles and additional clotting. The samples were assayed at 1:2 dilution.

### 4.10. Synovial Fluid (SF)

Synovial fluid was collected at 4 and 12 weeks after MSCs transplant using the following method: 300 µL of 0.9% NaCl was injected in 50 µL increments, medially to the patellar femoral ligament, and constantly aspirated by a syringe pump (Dose It—INTEGRA Biosciences^®^, Zizers, Switzerland) connected to a 24 G needle, laterally to the patellar femoral ligament. The samples were then centrifuged for 5 min at 10,000 rcf to remove cells and debris and stored at −80 °C. The protein concentration was measured using BCA assay (Pierce^TM^ BCA Protein Assay Kit—Thermo Fisher Scientific, Waltham, MA, USA) and normalized to 6 µg/µL (150 µg total) for the Luminex assay.

### 4.11. Cytokine/Chemokine Multiplex Assay

Cytokines/chemokines in the serum and SF samples were analyzed using the RECYTMAG-65K Millipore, MILLIPLEX MAP Rat Cytokine/Chemokine Magnetic Bead Panel (Merck KGaA, Darmstadt, Germany). The panel included: IL-1β, IL-6, IL-13, IL-10, TNFα, RANTES, MIP-1α, IL-4, IL-18, and MCP-1. Panel readings were conducted with the MAGPIX^®^ xMAP technology (Luminex^®^, Diasorin, Austin, TX, USA) following the manufacturer’s recommended protocol. Data were analyzed using MILLIPLEX^®^ Analyst v.5.1 (Merck KGaA, Darmstadt, Germany) software. Absolute values obtained with the Luminex assay (pg/mL) were standardized by square root (√(pg/mL)). To assess group changes in serum levels of cytokines/chemokines relative to controls, each individual data point was adjusted by subtracting the average value of either the pre-surgery (baseline control), SHAM surgery (negative control), or DMM–OA (positive control), depending on the specific comparison, resulting in a delta analysis. Non-detectable samples at individual time points were not included in the analysis.

### 4.12. Histology

The right knee joints were fixed in 4% paraformaldehyde and embedded in paraffin for coronal sectioning. Three to four sections were collected at the center of the knee’s medial tibial plateau (MTP) and stained with Safranin-O and Fast Green. The histological assessment of cartilage degeneration and synovium inflammation were conducted based on “The OARSI histopathology initiative” [58]. For cartilage scoring, the MTP was examined, while for synovium, the synovial lining opposite to the MTP was assessed. Matrix loss area (µm^2^) was measured by outlining the area of projected cartilage loss. Additionally, the area of matrix and GAG loss (µm^2^) were measured by delineating the area with projected cartilage loss, adjacent matrix with proteoglycan loss, and chondrocyte death. Synovium inflammation was assessed using a semiquantitative scoring system (0–4 grade). Measurements were performed using ImageJ2 v.2.1.0/1.53c software (https://imagej.net/). For all scoring systems, the average value between sections was considered for analysis.

### 4.13. Muscle Weight

After necropsy, the quadriceps, gastrocnemius and soleus, and tibialis anterior muscles were dissected (Figure S6C) from both hindlimbs and weighed individually on a precision balance. To reduce data variability in this analysis, all dissections were carried out by the same individual who had received prior training in the dissection technique. The muscle weights were then normalized by the body weight measured on the same day. The muscle in the contralateral limb muscle served as a morpho-functional control [59]. Therefore, muscle balance was determined by calculating the muscle weight ratio between the injured and contralateral hindlimbs.

### 4.14. Statistical Analysis

Statistical analysis was performed using Prism 9.2.0 (GraphPad Software, Boston, MA, USA). Data were tested for normality using Kolmogorov–Smirnov goodness-of-fit test. Student’s T-Test was used for comparing two means; one-way ANOVA with Fisher’s post hoc was used for comparing three independent means, and Tukey’s post hoc test for comparing four independent means; two-way ANOVA was used for group *x* time comparisons, and Sidák’s post hoc test was applied for multiple comparisons. Statistical significance was considered when α ≤ 0.05. Data are graphically represented by average, 95% confidence interval, and column scatter plots to indicate the sample size (N).

## 5. Conclusions

The injection of MSCs at a dose of 2 × 10^6^ cells/kg_bw_ induced an acute systemic inflammatory signaling, which was followed by a transient systemic anti-inflammatory response. Notably, each transplant route had different profiles of cytokines and chemokines expression. While MSCs showed the ability to migrate to the OA joint following IVt, the detection of viable bioluminescent cells persisted for a longer duration after IAt. Furthermore, the placebo control, which received intra-articular saline injection, exhibited a worse OA prognosis compared to the non-invasive control group. Finally, in the context of a low-grade OA model, a single injection of MSCs, whether via IAt or IVt, administered 9 weeks after the DMM surgery, did not inhibit AC degeneration when compared to a non-invasive control.

## Data Availability

Not applicable.

## Supplementary Materials

The following supporting information can be downloaded at: https://www.mdpi.com/article/10.3390/ijms242115543/s1.
