# Supplementary material for: Comparative Effects of Intra-Articular versus Intravenous Mesenchymal Stromal Cells Therapy in a Rat Model of Osteoarthritis by Destabilization of Medial Meniscus
Source: Int J Mol Sci. 2023 Oct 24;24(21):15543. doi: 10.3390/ijms242115543 (PMC10649289; doi:10.3390/ijms242115543)
Supplement: Supplementary file 1 [file ijms-24-15543-s001.zip › Supplementary Materials_review.pdf]

International Journal of
Molecular Sciences

MDPI

Supplementary Materials:

Methods S1. Anesthesia and pain monitoring

During the surgery, the animals were sedated using an inhalational anaesthesia equipment (SomnoSuite – Kent Scientific®, Torrington, CT, USA), with an anaesthetic mask. They were kept on a thermal blanket at 38 ºC (Physiosuite Right Temp – Kent Scientific®, Torrington, CT, USA), continuous air flow and isoflurane 3 % (Isoforine – Cristália®, Itapira, SP, Brazil). Before the procedure, the animals were sedated using 5 % isoflurane, and a preoperative analgesia protocol consisting of Tramadol 10 mg/mL + Dipyrone 200 mg/mL was administered at a dosage of 1.5 μL/g of the animal's weight intraperitoneally. All necessary aseptic procedures were performed before surgery. Euthanasia was conducted at the study endpoints within an adequate time frame of the animal model, ensuring there is no severe joint damage and associated distress. Analgesia and post-surgical follow-up were performed within the first 72 hours, and if any signs of pain were observed using the "facial pain scale" [53], the following analgesic protocol was applied:

- Mild pain (0.25 ≤ pain < 1): 0.25 μL/g* of animal in intramuscular injection; or 100 mg of dipyrone in 500 μL of gelatin;

- Moderate pain (1 ≤ pain < 1.5): 0.5 μL/g* of animal – intraperitoneal;

- Severe pain (pain ≥ 1.5): 1.0 μL/g* of animal – intraperitoneal;

- Surgical procedure: 1.5 μL/g* of anima – intraperitoneal;

*Analgesic solution: Tramadol Hydrochloride 10 mg/mL + Dipyrone 200 mg/mL.

Methods S2. Imaging Flow Cytometry

Cellular events were acquired using the standard configuration of the INSPIRE™ v.200.1 (Merck KGaA, Darmstadt, Germany) software for 1 hour. All events were classified in a focus region (gradient RMS_M01 - 40 to 86), followed by area vs. circularity XY dispersion (Area_M01; 100 to 1 000 μm versusaspectratio_M01; 1 to 0.6). M01 refers to the standard mask for the channel 01, using bright field images. The compensation matrix was automatically generated by the INSPIRE™ v.200.1 (Merck KGaA, Darmstadt, Germany) software software using cells labelled with only one lipophilic dye (DiL or DiD). Sample analyses and the application of the compensation matrix were performed using the IDEAS® 6.2 (Merck KGaA, Darmstadt, Germany). After defining masks for each channel, Ch01 - Brightfield; Ch03 - DiD and Ch11 – DiL, a new dispersion dot plot XY was generated relative to the fluorescence intensity Y = DiD versus X = DiL (Figure S4).

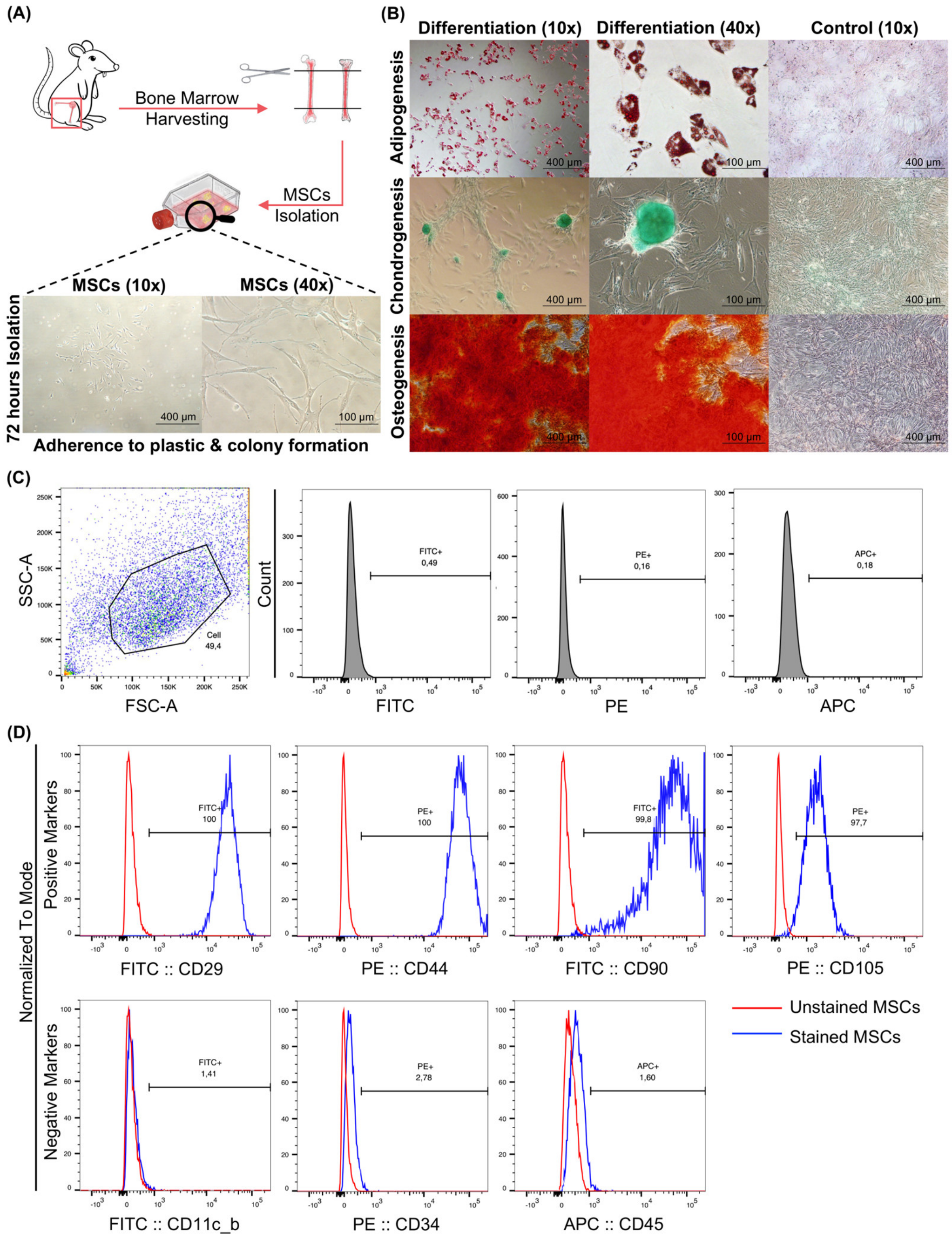

Figure S1. Isogenic rat bone marrow mesenchymal stromal cell (MSCs) Isolation. (A) Cells displaying a fibroblast like phenotype and adherence to plastic 72 hours after isolation (10x and 40x objective lenses). (B) Differentiation of MSCs in mesenchymal lineages (10x and 40x objective lenses). (C) From left to right, population gate and autofluorescence controls for unstained cells. (D) Characterization of positive (CD29, CD90, CD44, CD105) and negative (CD11c_b, CD45, CD34) surface markers by flow cytometry.

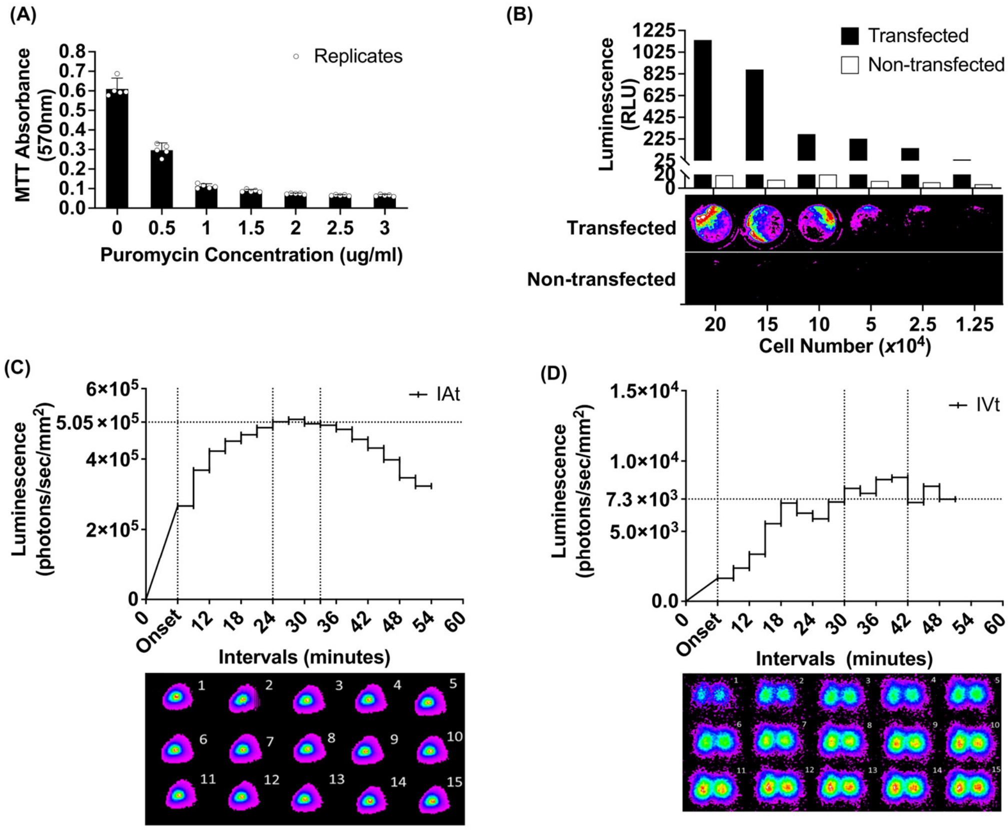

Figure S2. Mesenchymal stromal cells (MSCs) transfection and bioluminescence. (A) Puromycin toxicity curve for transduced cells selection measured by MTT absorbance assay. (B) Evaluation of bioluminescence efficiency in transfected cells compared to non-transfected cells, quantified in relative luminescence units (RLU), and imaged in a 96 wells culture plate (60× objective lens). (C-D) Time-lapse analysis of bioluminescence in transplanted cells 6 minutes after intraperitoneal injection of 150 mg/kg of D-luciferin. The intersection of vertical and horizontal dotted lines indicates the onset of signal collection and the optimal window for analysis. Rainbow colored images, from left or right, show in-vivo bioluminescent cellular signal in the kneejoint and lungs at each collection time point (1 to 15) after intra-articular (IAt; N=1) and intravenous (IVt; N=1) transplantation, respectively. Images were taken every 3 minutes for 45 minutes.

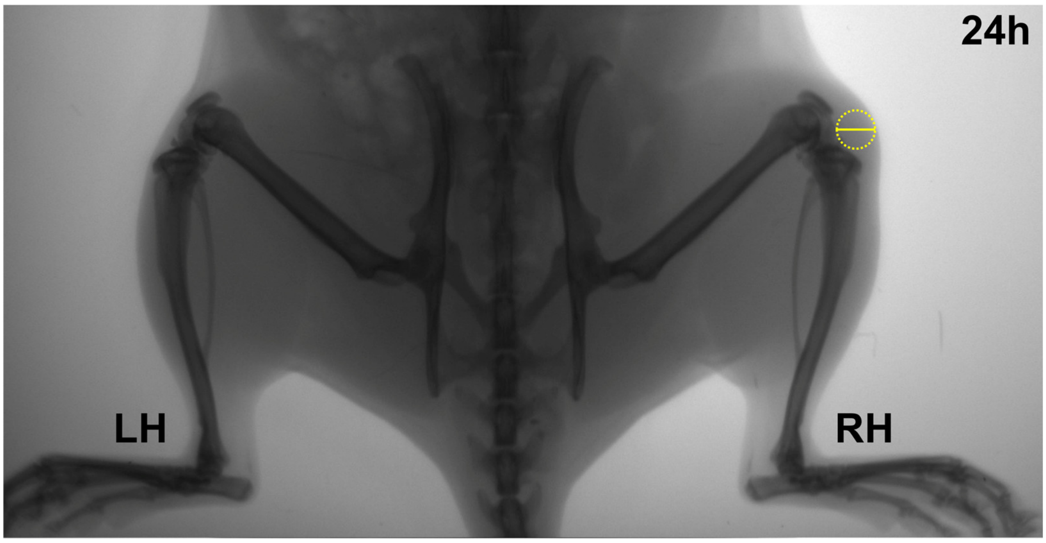

Figure S3. Knee swelling 24 hours after intra-articular mesenchymal stromal cell transplant. Radiography of both knees, in prone position. Yellow dashed circumference delineates the area between

the skin over the knee, the distal femur and proximal tibial epiphysis. Yellow solid line represents the diameter of this circumference which was used to the measurement of knee swelling. RH - right hind; LH - left hind.

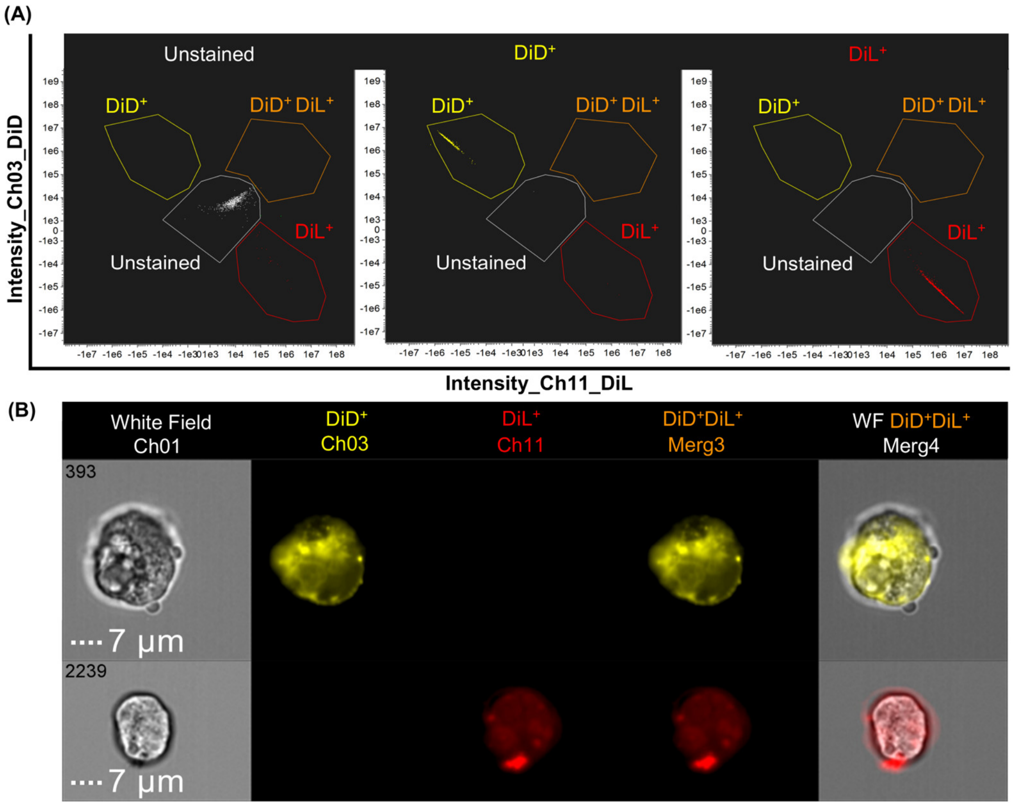

Figure S4. Mesenchymal stromal cells experimental control staining for Imaging Flow Cytometry. (A) Gating strategy dot plot for unstained cells (white dots), positive DiL+ cells (red dots) and positive DiD cells (yellow dots). (B) Images of cellular events with single DiD+ or DiL+ labeling (60× objective lens). White dotted line: 7 µm scalebar. IFC channels of labeled MSCs: DiD+ (yellow); DiL+ (red); DiL+ and DiD+ merge (orange).

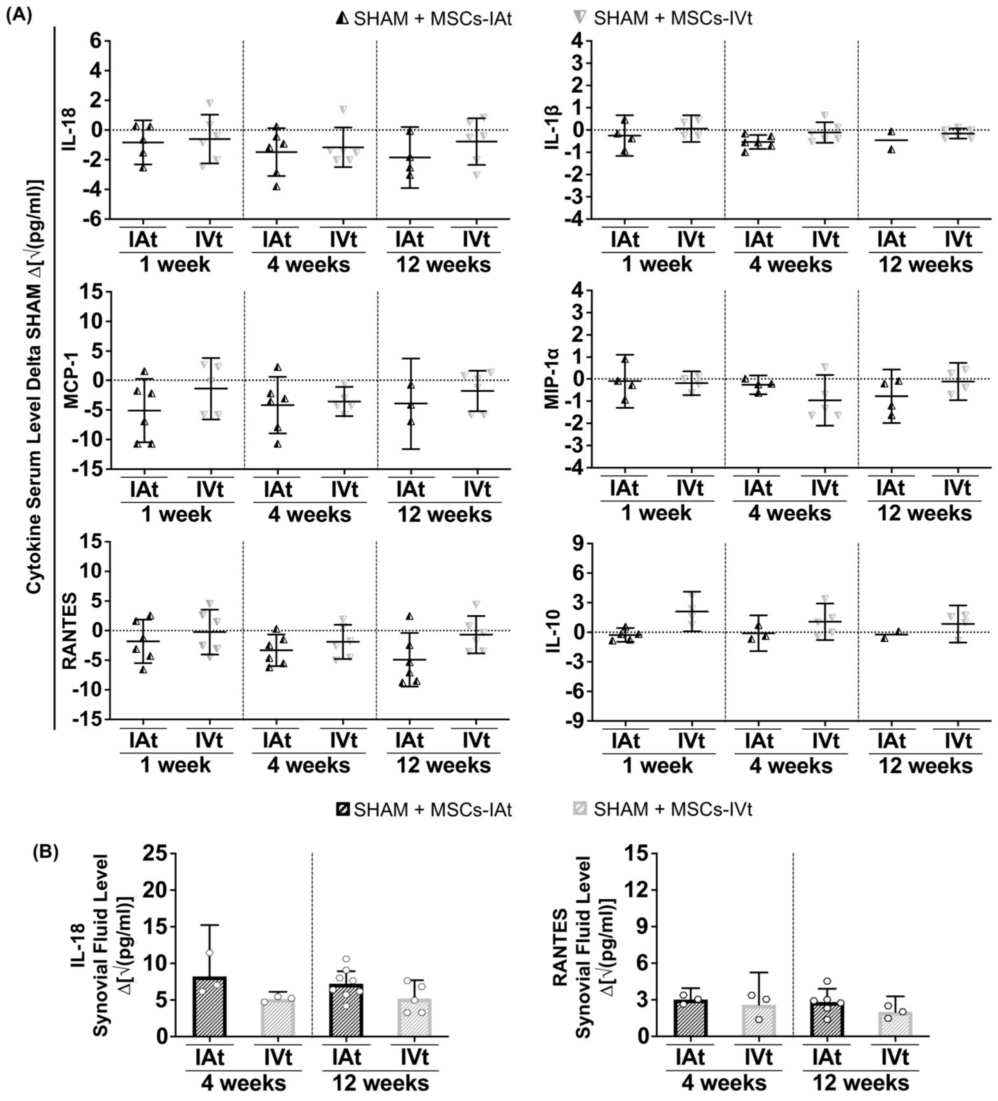

Figure S5. Long term immunomodulation at one (1w), four (4w) and twelve weeks (12w) after mesenchymal stroma cell (MSCs) intra-articular (IAt) or intravenous (IVt) transplantation in SHAM groups. (A) Serum and (B) synovial fluid levels of cytokines/chemokines measured by a multiplex bead assay. In the serum analysis, the horizontal dotted line represents the average serum level of each group at 9 weeks after Sham surgery (negative control), and data is expressed as the delta between the negative control and intervention groups. Vertical dashed lines isolate time points' comparisons. $p > 0.05$ for all statistical comparisons.

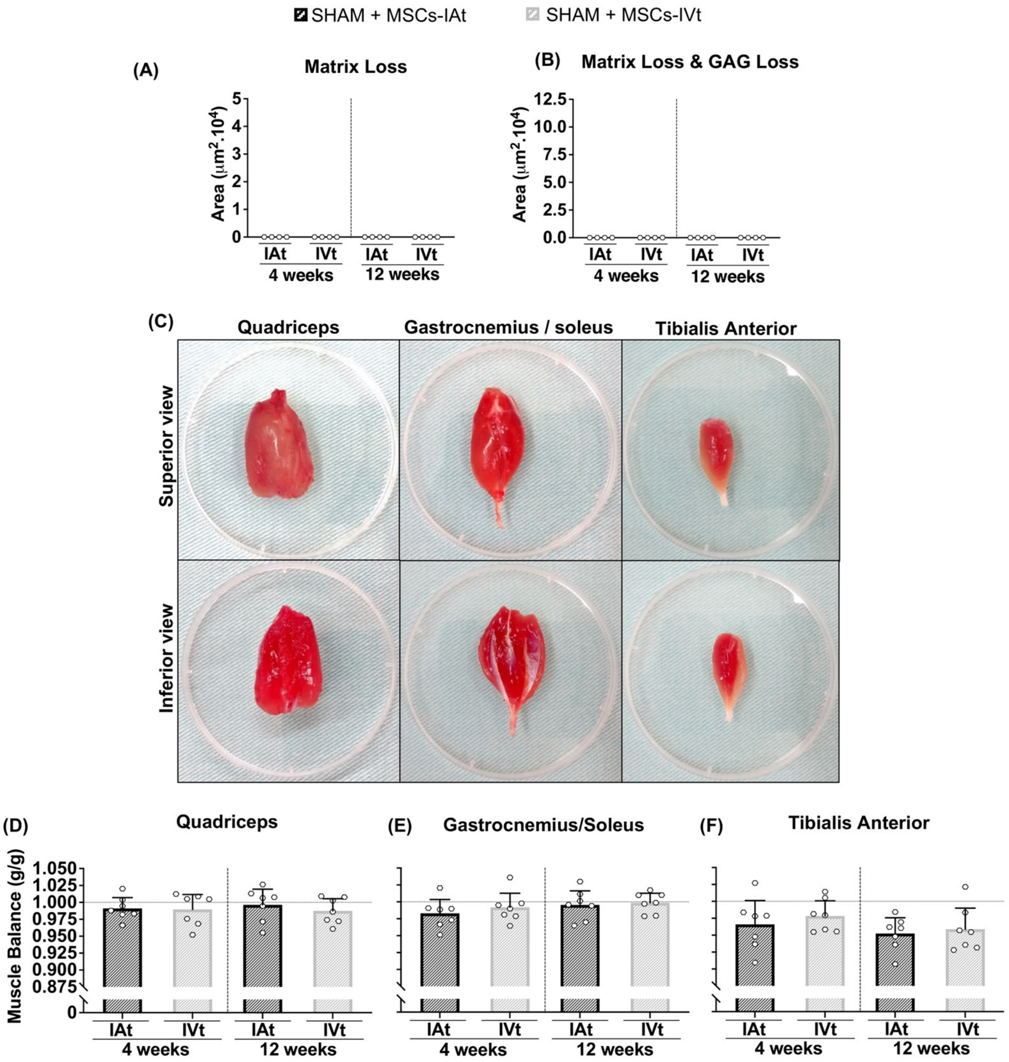

Figure S6. Muscle balance ratio and cartilage analysis after mesenchymal stromal cells (MSCs) intra-articular (IAt) or intravenous (IVt) treatment in SHAM groups. (A-B) Scoring of cartilage injury. (C) Hind limb muscle dissection for precision weighing. (D-F) Muscle balance ratio for quadriceps, gastrocnemius&soleus, and tibialis anterior between the SHAM and the contralateral limb joint (CL). Horizontal dotted line represents perfect muscle balance. Vertical dashed lines isolate timepoints comparisons. $p > 0.05$ for all statistical comparisons.

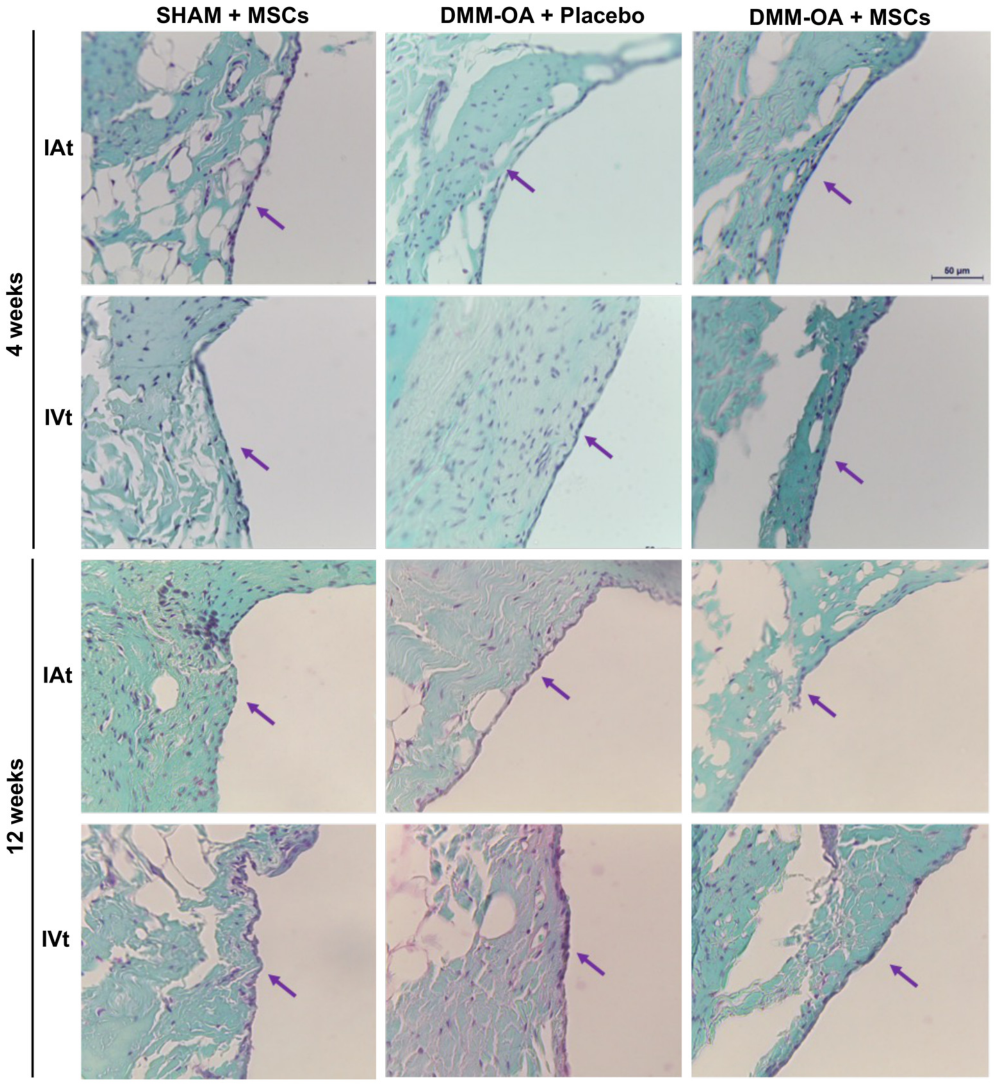

Figure S7. Synovial membrane histological assessment without important signs of synovial lining hyperplasia. Arrows indicate the synovial lining cells in purple.
